# Which factors influence psychiatric diagnosing in substance abuse treatment?

**DOI:** 10.1186/1752-4458-7-17

**Published:** 2013-06-06

**Authors:** Rolf Wynn, Anne Landheim, Ellen Hoxmark

**Affiliations:** 1 Division of Addictions and Specialized Psychiatric Services, University Hospital of North Norway, Tromsø, N-9291, Norway; 2 Department of Clinical Medicine, University of Tromsø, Tromsø, N-9037, Norway; 3 Innlandet Hospital Trust, Ottestad, N-2312, Norway

**Keywords:** Co-occurring disorders, Substance use disorders, Diagnostic practice, MINI-Plus

## Abstract

**Background:**

The importance of diagnosing and treating co-occurring psychiatric disorders among substance abusers in treatment has received much attention. The aim of this study was to investigate to which extent co-occurring psychiatric disorders are diagnosed in a clinical population of substance abusers, and which factors (including the use of MINI-Plus) that influence the diagnosing of co-occurring psychiatric disorders.

**Methods:**

Patients (*N* = 275) who received inpatient substance use treatment in five different units in Northern Norway participated in the study. The patients’ clinicians gave information on diagnoses given during the stay in the units, and whether a systematic diagnostic tool was used for the diagnosing (MINI-Plus). Predictors of independent co-occurring psychiatric disorders were examined utilizing hierarchical regression analysis.

**Results:**

One third of the patients were given an independent psychiatric diagnosis. Less than half of the patients were assessed using a diagnostic tool. The main predictor of diagnosing of independent psychiatric disorders was the use of the diagnostic tool MINI-Plus. Younger patients and patients that used less alcohol, were given independent psychiatric diagnoses more frequently.

**Conclusions:**

The number of co-occurring independent psychiatric diagnoses was lower compared to other studies using standardized diagnostic tools. The low number of patients assessed by such a tool, and the strong relationship between the use of such a tool and the diagnosing of co-occurring psychiatric disorders, suggest that the implementation of standardized diagnostic tools should be addressed in the units. Generally, patients suffering from substance use disorders should be systematically screened for other psychiatric disorders, in order to improve their treatment and health.

## Background

The diagnosing and treatment of co-occurring psychiatric disorders in substance abusers has received considerable attention [1,2]. A range of studies have found that psychiatric disorders are highly prevalent among substance abusers. Population studies indicate that psychiatric disorders and substance use disorders (SUDs) appear simultaneously among 30 – 40% of those with alcohol use disorders, and 40 – 50% of those with drug use disorders [3-8]. Studies of patients with SUDs in treatment report an even higher co-occurrence of psychiatric disorders [9-12]. Mood and anxiety disorders very often co-occur with SUDs [5,13,14]. Recent systematic reviews have revealed a strong association between depression and substance use [15-17]. A Norwegian study using the Composite International Diagnostic Interview [18] found that 85% of patients in a sample of substance abusers in treatment qualified for a current Axis 1 disorder in addition to the substance related disorder. The level of any anxiety disorders was 78% and the prevalence of major depression was 36%.

The identification of reliable and valid diagnoses of co-occurring psychiatric disorders in substance abusers may be problematic, especially as symptoms of SUD and non-SUD disorders may overlap [1,2]. The somewhat varying prevalence of SUD patients with co-occurring non-SUD diagnoses found in different studies [3-18] may at least in part reflect the complexity of reliable assessment. The nature of the relationship between substance use disorders and other psychiatric disorders is complex, and substance abuse may precede or follow other psychiatric disorders [19]. Psychiatric disorders may represent independent disorders, be related to acute intoxication or substance withdrawal states, or be the consequence of psychosocial stressors that many SUD patients experience, such as unemployment, poverty, loneliness, etc. [20,21]. Symptoms of anxiety, depression, mania and psychoses are all commonly induced by various substances and can be observed with chronic use as well as during specific substance-induced states; including intoxication and withdrawal. Studies have reported reductions in depressive symptoms during substance use treatment [22,23]. Reductions in depressive symptoms may also occur relatively rapidly after admission to both alcohol and opiate dependence treatment programs, as patients pass through acute intoxication and withdrawal states [24-26]. Also, symptoms of anxiety at admission to substance use treatment among alcohol dependent patients have been shown to spontaneously recede during treatment [27].

Failure to treat a concurrent psychiatric disorder reduces the likelihood that the treatment for SUD will be effective and can lead to negative consequences in a life time perspective [7,28,29]. It has been claimed that patients with psychiatric disorders often go undiagnosed and untreated for their psychiatric disorders through substance use treatment [30]. In a nationwide study of patients undergoing substance use treatment in Norway [31], only 25% of the patients were given a diagnosis according to the ICD-10 diagnostic system. An accurate diagnosis and a successful treatment of SUDs and co-occurring psychiatric disorders relies on a careful, comprehensive assessment [32], which can provide the necessary information for intervention and treatment planning. A careful and comprehensive assessment can also engage the patient and provide motivation to begin the process of change [33].

### The aim of the present study

This was the first study on substance use and co-occurring psychiatric disorders in one catchment area in Northern Norway including patients involving several units [34-37]. In this study, we wanted to investigate to which extent co-occurring psychiatric disorders were diagnosed in a clinical population of substance abusers. We were also interested in factors influencing diagnostic practice, and to which extent diagnostic practice varied with the variables that we had data on [38]. We hypothesized that there would be differences in diagnostic practice according to which extent a systematic diagnostic tool (i.e. the MINI-Plus) was used. We also hypothesized that a higher level of mental distress at admission to treatment predicted a diagnosis of a co-occurring psychiatric disorder. We wanted to address these hypotheses by investigating the following research questions:

1. What characterized patients in five different treatment units with regard to gender and age, substance use, independent psychiatric disorders, use of a diagnostic tool, and mental distress?

2. Did the use of a diagnostic tool (i.e. the MINI-Plus) predict the diagnosing of independent psychiatric disorders in this sample, when controlling for age, gender, use of substances, and mental distress?

## Methods

### Substance use treatment in Northern Norway

The five substance use treatment units participating in this study covered a population of 500 000 inhabitants in north Norway. All units were inpatient units at the University Hospital of Northern Norway. Inpatient care is the typical treatment setting for patients with SUD in northern Norway [39]. The Dual diagnosis ward offered specialized assessment and treatment of dual diagnoses and provided treatment up to six months. The Therapeutic community provided treatment up to 18 months according to a therapeutic community model. The Short-term unit was a detoxification unit that provided treatment up to six weeks. Long-term unit 1 and 2 provided general SUD inpatient treatment up to six months. All units treated both sexes, used a combination of group and individual therapy, and managed detoxification either directly or in collaboration with a detoxification unit. Treatment was composed of a combination of network-based approaches, psychotherapeutic and pharmacological treatments, but these components were given different emphasis in the various units. Characteristics of the patients within the different units are presented in Table 1.

**Table 1:** **Demographics**, **diagnoses**, **diagnostic practice**, **mental distress**, **and substance use according to unit**

**Unit**	**Dual diagnosis ward (*N*** **= 20)**	**Therapeutic community (*N*** **= 32)**	**Short-term unit (*N*** **= 87)**	**Long-term unit 1 (*N*** **= 68)**	**Long-term unit 2 (*N*** **= 68)**	**Total, mean value (*N*** **= 275)**
Mean age (SD)	27.75 (7.73)***	26.83 (6.32)***	39.17 (12.19)	43.38 (9.28)***	42.6 (10.75)**	38.79 (11.79)
Female	20% (4)	40.6% (13 )	26.4% (23)	26.5% (18)	25% (17)	27.3% (75)
MINI-Plus used	60% (12)	34.4% (11)	19.5% (17)***	45.6% (31)	69.1% (47)***	42.9% (118)
Independent psychiatric disorder	80% (16)***	34.4% (11)	19.5% (17)**	23.5% (16)	41.2% (28)	32% (88)
Anxiety disorder	40% (8)**	6.3% (2)	6.9% (6)*	16.2% (11)	20.6% (14)	14.9% (41)
Mood disorder	5% (1)	31.3% (10)**	9.2% (8)	1.5% (1)**	22.1% (15)**	12.7% (35)
Mean score HSCL-10 (SD)	2.86 (0.73)*	2.71 (0.43)*	2.71 (0.63)**	2.32 (0.8)**	2.35 (0.77)*	2.53 (0.73)
Mean score DUDIT (SD)	29.0 (9.84)***	35.28 (7.49)***	21.28 (15.83)	10.28 (13.81)***	12.56 (14.78)***	18.59 (16.14)
Mean score AUDIT (SD)	15.6 (9.7)	13.72 (10.9)*	14.4 (12.76)**	22.34 (10.02)***	19.74 (11.63)	17.69 (11.86)

*p < 0.05; **p < 0.01, ***p < 0.0001. T-tests and chi-square tests.

### Data collection procedures and participation rates

The study was based on a naturalistic design with measurements taken at admission to treatment and during treatment. All patients admitted to the units and considered competent to consent during the period September 2007 to May 2009 were given written and oral information about the study by a research collaborator working in each unit. Shortly following their consent to participate they responded to a questionnaire. During treatment, the patient’s clinician gave socio-demographic information (i.e. age and gender) and information about diagnostic procedures and outcome. Patients were paid compensation in the form of a cinema ticket or two lottery tickets (worth $8). The study was approved by the Regional Committee for Medical Research Ethics (P REK Nord 12/2006) and the Norwegian Social Science Data Services (NSD).

In the study period, 574 patients were admitted to the units. Patients who were considered not able to give an informed consent (*N* = 21) or whose hospital stay was too short to be included (*N* = 41) were not asked to participate. Of the patients considered relevant for the study (*N* = 512), 296 patients (58%) agreed to participate and signed an informed consent. Of these, 275 participants filled in the questionnaire at admission to treatment and are thus included in the analyses (73% men, mean age 39, range 18 – 79 years). More than 90% of the sample had a Norwegian origin.

### Outcome variable

All patients admitted to treatment in Norwegian hospitals are, at discharge, routinely given one or more diagnoses according to the ICD-10 system [40]. In this study, the diagnoses the participants were given were obtained from the patients’ clinicians. The clinicians used a standardized diagnostic tool (MINI-Plus), which a short time before the study started had been implemented for routine use in the units. However, not all clinicians used the MINI-Plus, and some preferred making a diagnostic assessment based on clinical judgement according to ICD-10 criteria. We were interested in the independent psychiatric diagnoses the participants were given that were not related to substance use. The participants were divided into two diagnostic categories: those who had obtained no other diagnosis than a SUD-diagnosis (F10-19) (*N* = 187), and those who had obtained one or more additional ICD-10 diagnoses (F20-99) (*N* = 88). Diagnostic category (i.e. the presence of a psychiatric diagnosis or not) was the outcome variable in the logistic regression analyses.

### Explanatory variables

Mental distress was measured at admission to treatment using a 10-item version of the Hopkins Symptom Check-List (HSCL-10) [41]. The HSCL-10 is a self-report questionnaire with a four point Likert scale, ranging from 1 (not at all) to 4 (extremely). The HSCL-10 is based on the SCL-90-R [42], and is composed of two out of the original nine factors (anxiety and depression) [43]. A mean item score was calculated and used as an index of general distress severity (Global Severity Index). A mean score of 1.85 or higher generally indicates a need of further assessment and possibly a need for psychiatric treatment [41].

Substance use was measured by the Alcohol Use Disorders Identification Test (AUDIT) [44] and the Drug Use Disorders Identification Test (DUDIT) [45]. The AUDIT is a widespread instrument measuring severity of alcohol use the past 12 months. It has 10 items with a scoring range from 0 to 40. The DUDIT is a parallel instrument to the AUDIT and is designed to identify persons with drug use disorders the past 12 months. It has 11 items with a scoring range from 0 to 44. Both the AUDIT and the DUDIT were used as continuous variables (i.e. without cut-off values).

The Norwegian National Client Assessment Form, which is routinely completed for all patients admitted to SUD treatment in Norway [46], was used to assess patients’ age and gender.

The patients’ clinicians provided extended information on the patients’ diagnostic assessment through a form developed especially for this study, including reporting the use of the diagnostic instrument MINI-Plus (Mini International Neuropsychiatric Interview-Plus). MINI-Plus is an extended version of the MINI [47] that is a structured interview for psychiatric disorders according to DSM-IV and ICD-10. MINI-Plus employs in addition different time frames for various disorders: current, past or lifetime [48].

### Statistical procedures

T-tests (for continuously scaled variables) and chi-squared tests (for dichotomous variables) were used to compare the participants in the different units, and later those who had obtained an independent psychiatric disorder with those who had not. The groups were compared on age, gender, whether or not they were assessed by the use of MINI-Plus, mental distress (measured by the HSCL-10) and substance use (measured by the AUDIT and the DUDIT). In the t-test analyses, the mean value of each unit was compared to the mean value of the other units.

The outcome variable was dichotomous, and a hierarchical binary logistic regression analysis was conducted to analyze associations between diagnosis and use of the diagnostic instrument MINI-Plus and mental distress, controlling for age, gender, and substance use (score on the AUDIT and the DUDIT). All analyses were conducted using SPSS 19.0.

## Results

### Descriptive statistics

Of the 275 participating patients, 88 (32%) were given an independent psychiatric disorder during treatment by the responsible clinician. 118 (42.9%) of the patients had been assessed by means of the MINI-Plus, the rest had been assessed by means of clinical judgement supported by ICD-10 criteria. The most common non-SUD diagnoses were anxiety disorders (15%) and mood disorders (13%). In addition, a few patients (4%) had other non-SUD diagnoses, such as ADHD, personality disorders, or schizophrenia. The most common SUD-diagnoses were alcohol dependence syndrome (45%), opioid dependency (23%), and dependency of other stimulants (12%). There were significant differences between the use of a systematic diagnostic tool (MINI-Plus) between the units, and also significant differences between the patients admitted to the different units (Table 1).

Compared to the overall mean values, the participants in the Dual diagnosis ward were younger, more often had an independent psychiatric disorder, more often had an anxiety disorder, and reported a higher score on HSCL-10 and a higher score on the DUDIT. Compared to the overall mean values, the participants from the Therapeutic community were younger, more often had a mood disorder, reported a higher score on the DUDIT and the HSCL-10, and a lower score on the AUDIT. Compared to the overall mean values, the MINI-Plus was less often used in the Short term unit, and the participants were less often given a diagnosis of an independent psychiatric disorder and less often given a diagnosis of an anxiety disorder. The participants also reported a higher score on the HSCL-10, and a lower score on the AUDIT. Compared to the overall mean values, the participants in Long term unit 1 were older. They were more seldom given a diagnosis of mood disorder, and reported a lower score on the HSCL-10 and the DUDIT and a higher score on the AUDIT. Compared to the overall mean values, the participants in the Long term unit 2 were also older. The MINI-Plus was used more often in this unit, and the participants were more often given a diagnosis of a mood disorder. The participants in Long term unit 2 reported a lower score on the HSCL-10 and the DUDIT.

Table 2 reports the results of the univariate analyses of the patients given an independent psychiatric disorder versus the patients given no independent psychiatric disorder. Compared to those who only had a SUD-diagnosis, patients who had an independent psychiatric disorder reported a higher score on the DUDIT, were more often female, and were more often assessed by a diagnostic instrument.

**Table 2:** Univariate analyses of patients who received an independent psychiatric disorder versus patients who did not

	***Total all N*** **=** ***275***	***Independent psychiatric disorder 32% *(*N*** **=** ***88*)**	***Only SUD*- *diagnosis 68% *(*N*** **=** ***187*)**	***p***
Age (*SD*)	38.8 (11.8)	34.2 (10.5)	41.0 (11.8)	*t* = 4.6**
Female	27.3% (75)	36.4% (32)	23% (43)	*x*^2^(1) = 5.4*
MINI-Plus used	118 (42.9%)	70.5% (62)	29.9% (56)	*x*^2^(1) = 40.1**
Mental distress at admission (HSCL-10)	2.53 (.73)	2.65 (.70)	2.48 (.73)	ns
AUDIT	17.69 (11.86)	15.68 (10.99)	18.64 (12.16)	ns
DUDIT	18.59 (16.14)	21.80 (15.67)	17.09 (16.18)	*t* = −2.3*

*p < .05; **p < .0001.

### Factors predicting diagnostic practice

A hierarchical logistic regression analysis was performed to identify predictors of a psychiatric diagnosis other than substance use disorder. Based on the univariate analyses, we investigated interactions between the use of MINI-Plus and the treatment units. A significant interaction between the use of MINI-Plus and the Short-Term Unit, Long Term Unit 1 and the Therapeutic community emerged. We thus decided to exclude all facilities as variables in the final analysis. The predictors were entered in three steps: 1) the use of a diagnostic instrument (MINI-Plus), 2) mental distress (HSCL-10), and 3) controlling for age, gender, and substance use (AUDIT and DUDIT). A total of 275 cases were analyzed and the full model significantly predicted a psychiatric diagnosis other than SUD (omnibus chi-square = 71.58, df = 6, *p* < 0.0001). The model accounted for between 22.9% and 32.1% of the variance in independent psychiatric disorders, with 88.8% of the non-additional diagnoses successfully predicted. Also, 50.0% of the additional diagnoses were successfully predicted. Overall, 76.4% of the predictions were successful. Table 3 gives coefficients, the Wald statistics, and probability values for each of the predictor variables. This shows that the use of the diagnostic instrument MINI-Plus predicted the setting of an independent psychiatric disorder, in addition to the control variables lower age and a lower use of alcohol as measured by the AUDIT.

**Table 3:** Logistic regression analysis of factors predicting diagnostic practice

	***B***	***SE***	***Wald***	***p***	***OR***	***95% CI for OR***
MINI-Plus used	1.970	.317	38.554	.0001	7.172	3.851-13.358
Age	-.057	.017	11.585	.001	.945	.915-.976
Use of alcohol (AUDIT)	-.030	.015	4.031	.045	.971	.943-.999

## Discussion

This study of diagnostic practice in five substance use units in northern Norway showed that one third of the patients were given an independent psychiatric diagnosis.

The main predictor of the diagnosing of independent psychiatric diagnoses was the use of a systematic diagnostic tool (MINI-Plus). Younger age and less use of alcohol as measured by the AUDIT [44] were also associated with independent psychiatric diagnoses.

We used diagnoses of independent psychiatric disorders as the outcome measure, as we were interested in how clinicians diagnosed patients with substance use disorders. Previous research has shown that substance use disorders and other psychiatric disorders to a great extent appear simultaneously [3-5]. We found a lower number of independent psychiatric diagnoses in our study compared to previous research using standardized diagnostic instruments [18]. This finding is probably due to the fact that the diagnoses in our study most often were based on clinical judgement. In substance use treatment, naturally, the main focus is on substance use disorders, and there could be a tendency to overlook co-occurring psychiatric disorders [32]. This finding is in line with previous research that reports that patients with psychiatric disorders often go undiagnosed and untreated through substance use treatment [30]. The most common non-SUD diagnoses were anxiety and mood disorders, as found in previous studies. The proportion of patients with these diagnoses was nevertheless much lower compared to other studies [18]. In one other Norwegian study, 78% of the patients had been given a diagnosis of a mood disorder and 36% had received a diagnosis of major depression [18]. In our study, only 15% had been given a diagnosis of an anxiety disorder and only 13% a diagnosis of a mood disorder.

This study revealed significant differences between the different units, concerning most of the variables studied (Table 1). Independent psychiatric disorders were significantly more often identified in the Dual diagnosis ward, and less often in the Short-term unit. This is probably not surprising given that the Dual diagnosis ward is specialized in treating exactly such a patient group, meaning that patients with dual diagnoses are channeled to this ward and that there is a stronger focus on dual diagnoses in this ward. On the other hand, independent mood disorders were identified in only 5% of the patient in this unit. This is more seldom than in the general population [4]. The Short-term ward deals more with detoxification and refers patients to other units for further assessment and treatment. They also used the standardized diagnostic tool MINI-Plus significantly less often than the other units, probably due to time constraints and a high proportion being in a phase of detoxification. A more surprising and less obvious fact was to which degree the proportion of patients that were given the most common independent psychiatric disorders (anxiety and mood disorders) varied between the units. Anxiety disorders were overall the most common independent psychiatric disorders. This is consistent with other studies, even though the proportion was much lower here than in other studies [18]. Diagnoses of anxiety disorders were more often used in the Dual diagnosis ward, and less often in the Short-term unit. This pattern follows the pattern of independent psychiatric disorders as such. Mood disorders were more often diagnosed in the Therapeutic community and in Long-term unit 2, and less often in Long-term unit 1. Also, only one of the patients in the Dual diagnosis ward was given a diagnosis of mood disorder. Clinicians at Long-term unit 2 used the MINI-Plus significantly more often than the clinicians in the other units. The two Long-term units only differed when it came to the use of MINI-Plus and the diagnosing of mood disorders. It is difficult to understand the varying use of MINI-Plus as anything but an expression of different cultures or treatment philosophies in the various units [49].

As previously reported from this study, the level of mental distress according to HSCL-10 was high in this sample [34]. The cut-off point generally agreed on is 1.85 [41]. In this sample, the mean score varied between 2.32 (Long-term unit 1) and 2.86 (the Dual diagnosis ward), with 2.53 as mean score in the entire sample. The level of mental distress reported was significantly higher in the Dual diagnosis ward, the Therapeutic community and the Short-term unit, and significantly lower in the two Long-term units. This pattern is comparable with the pattern of use of alcohol and other substances. A higher level of mental distress at admission to treatment was related to less heavy use of alcohol and more heavy use of other substances. This is consistent with previous research that finds a greater incidence of independent psychiatric disorders among patients with drug use disorders compared to patients with alcohol use disorders [10].

In this study, younger patients more often received a diagnosis of an independent psychiatric disorder in addition to their substance abuse diagnosis. We also found an association between less heavy use of alcohol as measured by the AUDIT [44] and independent psychiatric disorders. We did not find that drug use as measured by the DUDIT predicted the setting of non-SUD disorders. While some previous research has suggested that psychiatric disorders appear less often for those with an alcohol related disorder compared with those with drug use disorders [3-6,8,50], this was not the case in our study. Previous publications from the present study have shown that participants with alcohol related disorders in this sample were older than those with drug use disorders [34]. The variable gender was not significant, which is interesting since prior studies have found that the gender of the patient is an important factor with respect to both the distribution of mental disorders [10] and diagnostic practice [38].

Level of mental distress had no significant impact on the diagnosing of independent psychiatric disorders. One would expect level of mental distress to be an important factor, and that patients with high levels of mental distress would be more likely to receive additional psychiatric diagnoses, especially diagnoses related to depression and anxiety. It is possible that the level of mental distress at admission to treatment in this sample was so high that it did not differentiate between those who should receive a psychiatric diagnosis and those who should not.

Using the MINI-Plus increased the chance of the patient receiving a second diagnosis. Structured interviews have been shown to increase the diagnostic validity of SUD-diagnoses compared to clinical judgements, but the validity of structured interviews for detecting co-occurring psychiatric diagnoses has been questioned [51,52]. In the present study, the clinicians who chose to use the MINI-Plus had likely suspected that the patients had co-occurring psychiatric disorders. We believe that psychiatric disorders in this population often are overlooked. The findings that patients admitted to a specific ward and/or subjected to investigation by the MINI-Plus more often received additional psychiatric diagnoses, may support this idea.

### Strengths and limitations

This is one of only a few studies in Norway that have surveyed independent psychiatric disorders among substance abusers in treatment, and the first in the northern part of Norway. Our study covered the range of treatment facilities that is most commonly offered substance abusers in the area. Northern Norway is a relatively well defined catchment area where most of the treatment is given in inpatient settings.

The present study is subject to a number of limitations. The participation rate was 58%. While the rate is not particularly low for this type of study, it might nevertheless impact the representativity of the findings. In addition, the study sample was selected from five different units for inpatient SUD treatment. The units differed substantially - one unit was primarily concerned with detoxification, one unit focused on the assessment of dual diagnosis patients, and three units offered a more goal directed SUD treatment. As type of treatment unit was excluded from the regression analysis due to interaction effects, we are unable to make any claims about the importance of type of treatment unit on diagnostic practice. We also lack information on the clinicians that made the assessments. There is, therefore, some heterogeneity within the sample. A further limitation is that the study lacks an untreated control condition, although this is extremely difficult to construct in this study setting. On the other hand, multisite, prospective studies like the present can investigate treatment outcomes in existing services and under actual clinical circumstances, and thereby show a high external validity and allow for a generalization of the findings to clinical settings [53].

## Conclusions

One third of the patients were given an independent psychiatric diagnosis, which was low compared to other studies using standardized diagnostic tools. In this study, less than half of the patients were assessed using a diagnostic tool. The main predictor of diagnosing of independent psychiatric disorders was the use of the diagnostic tool MINI-Plus. Younger patients and patients that used less alcohol, were given independent psychiatric diagnoses more frequently. The low number of patients assessed by a standardized diagnostic tool, and the strong relationship between the use of a diagnostic tool and the diagnosing of co-occurring diagnoses, suggest that the implementation of standardized and validated diagnostic tools should be addressed in the units involved in the study. In order to provide good quality care, SUD-patients should be systematically examined for non-SUD disorders.

## Abbreviations

SUD: Substance use disorder.

## Competing interests

The authors declare that they have no competing interests.

## Authors’ contributions

EH and RW designed the study. EH was responsible for collecting the data and performed the statistical procedures. All authors contributed to the analysis of the data. EH drafted the manuscript. All authors revised the manuscript and read and approved the final manuscript.

## References

[B1] Mueser KT Noordsy DL Drake RE Fox L Integrated treatment for dual disorders: a guide to effective practice 2003 New York: The Guildford Press

[B2] Kelly TM Daley DC Douaihy AB Treatment of substance abusing patients with comorbid psychiatric disorders Addict Behav 2012 37 11 24 10.1016/j.addbeh.2011.09.010 21981788 PMC3196788

[B3] Regier DA Farmer ME Rae DS Locke BZ Keith SJ Judd LL Comorbidity of mental disorders with alcohol and other drug abuse. Results from the Epidemiologic Catchment Area (ECA) Study JAMA 1990 264 2511 2518 10.1001/jama.1990.03450190043026 2232018

[B4] Alonso J Angermeyer MC Bernert S Bruffaerts R Brugha TS Bryson H 12-Month comorbidity patterns and associated factors in Europe: results from the European Study of the Epidemiology of Mental Disorders (ESEMeD) project Acta Psychiatr Scand 2004 109 28 37 10.1111/j.1600-0047.2004.00328.x 15128385

[B5] Grant BF Stinson FS Dawson DA Chou SP Dufour MC Compton W Prevalence and co-occurrence of substance use disorders and independent mood and anxiety disorders: results from the National Epidemiologic Survey on Alcohol and Related Conditions Arch Gen Psychiatry 2004 61 807 816 10.1001/archpsyc.61.8.807 15289279

[B6] Kessler RC Chiu WT Demler O Walters EE Prevalence, severity, and comorbidity of 12-Month DSM-IV disorders in the National Comorbidity Survey Replication Arch Gen Psychiatry 2005 62 617 627 10.1001/archpsyc.62.6.617 15939839 PMC2847357

[B7] Hasin D Liu X Nunes E McCloud S Samet S Endicott J Effects of major depression on remission and relapse of substance dependence Arch Gen Psychiatry 2002 59 375 380 10.1001/archpsyc.59.4.375 11926938

[B8] Jané-Llopis E Matytsina I Mental health and alcohol, drugs and tobacco: a review of the comorbidity between mental disorders and the use of alcohol, tobacco and illicit drugs Drug Alcohol Rev 2006 25 515 536 10.1080/09595230600944461 17132571

[B9] Ross HE Glaser FB Germanson T The prevalence of psychiatric disorders in patients with alcohol and other drug problems Arch Gen Psychiatry 1988 45 1023 1031 10.1001/archpsyc.1988.01800350057008 3263100

[B10] Landheim AS Bakken K Vaglum P Sammensatte problemer og separate systemer. Psykiske lidelser blant rusmisbrukere til behandling i russektoren (Complex problems and separate systems. Psychiatric illness among substance abusers in substance abuse treatment) Nor Epidemiol 2002 12 309 318

[B11] Wynn R Prior psychotic episodes among patients in a substance abuse clinic J Subst Use 2007 12 127 132 10.1080/14659890601178675

[B12] Verheul R Patients with addiction and personality disorder: treatment outcomes and clinical implications Curr Opin Psychiatry 2007 20 67 71 10.1097/YCO.0b013e328011740c 17143086

[B13] O’Brien CP Charney DS Lewis L Cornish JW Post RM Woody GE Priority actions to improve the care of persons with co-occurring substance abuse and other mental disorders: A call to action Biol Psychiatry 2004 56 703 713 10.1016/j.biopsych.2004.10.002 15556110

[B14] Hailemariam S Tessema F Asefa M Tadesse H Tenkolu G The prevalence of depression and associated factors in Ethiopia: findings from the National Health Survey Int J Ment Health Syst 2012 6 23 10.1186/1752-4458-6-23 23098320 PMC3511231

[B15] Marshall BDL Werb D Health outcomes associated with methamphetamine use among young people: a systematic review Addiction 2010 105 991 1002 10.1111/j.1360-0443.2010.02932.x 20659059

[B16] Conner KR Pinquart M Holbrook AP Meta-analysis of depression and substance use and impairment among cocaine users Drug Alcohol Depend 2008 98 13 23 10.1016/j.drugalcdep.2008.05.005 18585871 PMC2570759

[B17] Conner KR Pinquart M Gamble SA Meta-analysis of depression and substance use among individuals with alcohol use disorders J Subst Abuse Treat 2009 37 127 137 10.1016/j.jsat.2008.11.007 19150207 PMC4864601

[B18] Landheim AS Bakken K Vaglum P Gender differences in the prevalence of symptom disorders and personality disorders among poly-substance abusers and pure alcoholics. Substance abusers treated in two counties in Norway Eur Addict Res 2003 9 8 17 10.1159/000067732 12566793

[B19] Goodwin RD Stein DJ Anxiety disorders and drug dependence: Evidence on sequence and specificity among adults Psychiatry Clin Neurosci 2013 67 167 73 10.1111/pcn.12030 23581868 PMC3701305

[B20] Myrick H Brady K Current review of the comorbidity of affective, anxiety, and substance use disorders Curr Opin Psychiatry 2003 16 261 270

[B21] Nunes EL Levin FR Treatment of depression in patients with alcohol and other depression JAMA 2004 291 1887 1896 10.1001/jama.291.15.1887 15100209

[B22] Carroll KM Rounsaville BJ Gordon LT Nich C Jatlow P Bisighini RM Psychotherapy and pharmacotherapy for ambulatory cocaine abusers Arch Gen Psychiatry 1994 51 177 187 10.1001/archpsyc.1994.03950030013002 8122955

[B23] Husband SD Marlowe DB Lamb RJ Iguchi MY Bux DA Kirby KC Decline in self-reported dysphoria after treatment entry in inner-city cocaine addicts J Consult Clin Psychol 1996 64 221 224 8907102 10.1037//0022-006x.64.1.221

[B24] Brown SA Schuckit MA Changes in depression among abstinent alcoholics J Stud Alcohol 1988 49 412 417 3216643 10.15288/jsa.1988.49.412

[B25] Strain EC Stitzer ML Bigelow GE Early treatment time course of depressive symptoms in opiate addicts J Nerv Ment Dis 1991 179 215 221 10.1097/00005053-199104000-00007 2007892

[B26] Nunes EL Sullivan M Levin FR Treatment of depression in patients with opiate dependence Biol Psychiatry 2004 56 793 802 10.1016/j.biopsych.2004.06.037 15556125

[B27] Brown SA Irwin M Schuckit M Changes in anxiety among abstinent male alcoholics J Stud Alcohol 1991 52 55 61 1994124 10.15288/jsa.1991.52.55

[B28] Greenfield SF Weiss RD Muenz LR Vagge LM Kelly JF Bello LR The effect of depression on return to drinking: A prospective study Arch Gen Psychiatry 1998 55 259 265 10.1001/archpsyc.55.3.259 9510220

[B29] Fridell M Hesse M Psychiatric severity and mortality in substance abusers: A 15-year follow-up of drug users Addict Behav 2006 31 559 565 10.1016/j.addbeh.2005.05.036 15967584

[B30] Chan YF Dennis ML Funk RR Prevalence and comorbidity of major internalizing and externalizing problems among adolescents and adults presenting to substance abuse treatment J Subst Abuse Treat 2008 34 14 24 10.1016/j.jsat.2006.12.031 17574804 PMC2238174

[B31] Gråwe R Ruud T Rus og psykiske lidelser i psykisk helsevern for voksne. (Substance use and mental disorders in adult mental treatment in Norway) (Rep. No. STF78 A06003) 2006 SINTEF Helse: Oslo/Trondheim

[B32] Weiss RD Identifying and diagnosing co-occurring disorders CNS Spectr 2008 13 4 6 19317016 10.1017/s1092852900002893

[B33] Wynn R Karlsen K Lorntzsen B Bjerke TN Bergvik S Users and GPs causal attributions of illegal substance use: An exploratory interview study Patient Educ Couns 2009 76 227 232 10.1016/j.pec.2009.01.001 19201142

[B34] Hoxmark E Nivison M Wynn R Predictors of mental distress among substance abusers receiving inpatient treatment Subst Abuse Treat Prev Policy 2010 5 15 10.1186/1747-597X-5-15 20609222 PMC2907362

[B35] Hoxmark E Benum V Friborg O Wynn R Reduction in mental distress among substance users receiving inpatient treatment Int J Ment Health Syst 2010 4 30 10.1186/1752-4458-4-30 21122161 PMC3014872

[B36] Sletteng R Harnang AK Hoxmark E Aslaksen PM Friborg O Wynn R A Psychometric study of the drug use disorders identification test - Extended in a Norwegian sample Psychol Rep 2011 109 663 674 10.2466/03.09.13.15.PR0.109.5.663-674 22238864

[B37] Hoxmark E Wynn TN Wynn R Loss of activities and its effect on the well-being of substance abusers Scand J Occup Ther 2012 19 78 83 10.3109/11038128.2011.552120 21299367

[B38] Høye A Rezvy G Hansen V Olstad R The effect of gender in diagnosing early schizophrenia Soc Psychiatry Psychiatr Epidemiol 2006 41 549 555 10.1007/s00127-006-0066-y 16699815

[B39] Andreassen M Seloter PB Pasientstrømmen til tverrfaglig spesialisert rusbehandling i Nord-Norge (Admission to multidisciplinary specialized substance abuse treatment in Northern Norway) 2005 Narvik, Norway: Nordnorsk kompetansesenter-Rus

[B40] World Health Organization The ICD-10 classification of mental and behavioural disorders: Clinical descriptions and diagnostic guidelines 1992 10 Geneva: World Health Organisation

[B41] Strand BH Dalgard OS Tambs K Rognerud M Measuring the mental health status of the Norwegian population: A comparison of the instruments SCL-25, SCL-10, SCL-5 and MHI-5 (SF-36) Nord J Psychiatry 2003 57 113 118 10.1080/08039480310000932 12745773

[B42] Derogatis LR Lipman RS Rickels K Uhlenhuth EH Covi L The Hopkins Symptom Checklist (HSCL): a self-report symptom inventory Behav Sci 1974 19 1 15 10.1002/bs.3830190102 4808738

[B43] Lipman RS Covi L Shapiro AK The Hopkins Symptom Checklist (HSCL): Factors derived from the HSCL-90 J Affect Disord 1979 1 9 24 10.1016/0165-0327(79)90021-1 162184

[B44] Saunders JB Aasland OG Babor TF De La Fuente JR Grant M Development of the Alcohol Use Disorders Identification Test (AUDIT): WHO collaborative project on early detection of persons with harmful alcohol consumption–II Addiction 1993 88 791 804 10.1111/j.1360-0443.1993.tb02093.x 8329970

[B45] Berman AH Bergman H Palmstierna T Schlyter F Evaluation of the Drug Use Disorders Identification Test (DUDIT) in criminal justice and detoxification settings and in a Swedish population sample Eur Addict Res 2005 11 22 31 10.1159/000081413 15608468

[B46] Iversen E Lauritzen G Skretting A Skutle A Klientkartleggingsdata: rapport for 2008 (Client data: report 2008) 2009 Bergen, Norway: Stiftelsen Bergensklinikkene and Statens institutt for rusmiddelforskning

[B47] Sheehan DV Lecrubier Y Sheehan KH Amorim P Janavs J Weiller E The Mini-International Neuropsychiatric Interview (M.I.N.I): The development and validation of a structured diagnostic psychiatric interview for DSM-IV and ICD-10 J Clin Psychiatry 1998 59 22 33 9881538

[B48] Gunter TD Arndt S Wenman G Allen J Loveless P Sieleni B Frequency of mental and addictive disorders among 320 men and women entering the Iowa Prison System: Use of the MINI-Plus J Am Acad Psychiatry Law 2008 36 27 34 18354120

[B49] Hoxmark E Wynn R Health providers’ descriptions of the significance of the therapeutic relationship in treatment of patients with dual diagnoses J Addict Nurs 2010 21 187 193 10.3109/10884602.2010.520170

[B50] Hasin DS Stinson FS Ogburn E Grant BF Prevalence, correlates, disability, and comorbidity of DSM-IV alcohol abuse and dependence in the United States: Results from the National Epidemiologic Survey on Alcohol and Related Conditions Arch Gen Psychiatry 2007 64 830 842 10.1001/archpsyc.64.7.830 17606817

[B51] Kranzler HR Kadden RM Burleson JA Babor TF Apter A Rounsaville BJ Validity of psychiatric diagnoses in patients with substance use disorders: Is the interview more important than the interviewer? Compr Psychiatry 1995 36 278 288 10.1016/S0010-440X(95)90073-X 7554872

[B52] Langås AM Malt UF Opjordsmoen S Comorbid mental disorders in substance users from a single catchment area - a clinical study BMC Psychiatry 2011 11 25 10.1186/1471-244X-11-25 21314980 PMC3042931

[B53] Gossop M Marsden J Stewart D Remission of psychiatric symptoms among drug misusers after drug dependence treatment J Nerv Ment Dis 2006 194 826 832 10.1097/01.nmd.0000244483.17443.0e 17102706

